# FLA-UNet: feature-location attention U-Net for foveal avascular zone segmentation in OCTA images

**DOI:** 10.3389/frai.2025.1463233

**Published:** 2025-07-17

**Authors:** Wei Li, Li Cao, He Deng

**Affiliations:** ^1^School of Electrical and Electronic Engineering, Wuhan Polytechnic University, Wuhan, China; ^2^School of Computer Science and Technology, Wuhan University of Science and Technology, Wuhan, China

**Keywords:** optical coherence tomography angiography (OCTA), foveal avascular zone (FAZ) segmentation, feature-location attention, joint loss function, U-Net

## Abstract

**Introduction:**

Since optical coherence tomography angiography (OCTA) is non-invasive and non-contact, it is widely used in the study of retinal disease detection. As a key indicator for retinal disease detection, accurate segmentation of foveal avascular zone (FAZ) has an important impact on clinical application. Although the U-Net and its existing improvement methods have achieved good performance on FAZ segmentation, their generalization ability and segmentation accuracy can be further improved by exploring more effective improvement strategies.

**Methods:**

We propose a novel improved method named Feature-location Attention U-Net (FLA-UNet) by introducing new designed feature-location attention blocks (FLABs) into U-Net and using a joint loss function. The FLAB consists of feature-aware blocks and location-aware blocks in parallel, and is embed into each decoder of U-Net to integrate more marginal information of FAZ and strengthen the connection between target region and boundary information. The joint loss function is composed of the cross-entropy loss (CE loss) function and the Dice coefficient loss (Dice loss) function, and by adjusting the weights of them, the performance of the network on boundary and internal segmentation can be comprehensively considered to improve its accuracy and robustness for FAZ segmentation.

**Results:**

The qualitative and quantitative comparative experiments on the three datasets of OCTAGON, FAZID and OCTA-500 show that, our proposed FLA-UNet achieves better segmentation quality, and is superior to other existing state-of-the-art methods in terms of the MIoU, ACC and Dice coefficient.

**Discussion:**

The proposed FLA-UNet can effectively improve the accuracy and robustness of FAZ segmentation in OCTA images by introducing feature-location attention blocks into U-Net and using a joint loss function. This has laid a solid theoretical foundation for its application in auxiliary diagnosis of fundus diseases.

## 1 Introduction

With the rapid development and popularization of medical imaging equipment, the imaging technology has been widely used in clinical practice, and become an indispensable auxiliary means to carry out disease diagnosis, surgical planning, prognosis assessment and so on. Optical coherence tomography angiography (OCTA) (Kashani et al., 2017) is a new non-invasive fundus imaging technology, which uses light interference to obtain vascular structure and blood flow information, and provide high resolution vascular imaging. In recent years, OCTA has been widely used in clinical diagnosis of various eye diseases, such as macular region disease, diabetic retinopathy, and retinal vascular obstruction. These eye diseases are related to the size and morphological changes of foveal avascular zone (FAZ) (Chui et al., 2012), which is surrounded by continuous capillary plexus of the retina, and does not have any capillary structure itself. It is an important area for the formation of fine visual function. The changes in its shape and surrounding capillary density reflect the degree of ischemia of the macula, and are closely related to retinal vascular diseases, such as diabetic retinopathy and retinal venous obstruction. For the three eye-related conditions, namely normal, diabetes and myopia (Balaji et al., 2020), diabetic eyes have a statistically significant increase in FAZ area compared to normal eyes. Similarly, the FAZ area increases and the blood vessel diameter decreases in myopia, especially in high myopia. Therefore, the changes in the area and morphology of FAZ can provide an important basis for clinical diagnosis of diabetes and myopia. The accurate segmentation of FAZ in OCTA images is crucial for diagnosis of fundus diseases.

In early days, many classical methods are proposed for FAZ segmentation. For example, the methods based on threshold segmentation (Liu et al., 2022), region growth (Ghassemi and Mirzadeh, 2007) and morphological operation (Yang et al., 2016) can be used to segment FAZ, by setting appropriate thresholds or using local features of images. However, these methods may have some limitations when dealing with complex image conditions. To further improve the segmentation performance, some methods based on traditional machine learning algorithms are proposed, such as Markov Random Fields (MRF) (Bourennane, 2010) and Support Vector Machine (SVM) (Alam et al., 2019), where hand-crafted features and traditional classifiers are used for segmentation. Nevertheless, the segmentation accuracy is usually limited by the selection of features and the capability of classifiers.

In recent years, with the development of deep learning technology, fundus image segmentation methods based on deep learning have achieved great success. A typical example is the segmentation method using U-Net (Ronneberger et al., 2015; Sherwani and Gopalakrishnan, 2024), which is a kind of full convolutional network with simple structure and beneficial effect. As this method processes the whole image in the same way and cannot give different attentions to different areas, various improvement methods based on U-Net are proposed later. The introduction of attention mechanism (Chen et al., 2022) in network models is one of the most effective ways, which can improve the accuracy and stability of segmentation by focusing on FAZ. These methods are often implemented by adding attention branches to models and adjusting the weights of features in channel and spatial dimensions, respectively. For instance, the channel attention branch can globally model the channels on feature maps and adjust the importance of each channel according to task requirements, to better express attention on FAZ. The spatial attention branch can consider the position relationship between pixels in spatial dimension to adjust the weight of each pixel in global feature maps, so as to accurately segment FAZ.

In addition to the improvement of the network structure, another improvement point is adopting a more appropriate loss function to optimize the model parameters, so as to improve the performance of the network model. For example, a hybrid loss function is used in DT-Net to improve the accuracy of retinal vessel segmentation (Jia et al., 2023), and a joint loss function is used in a multi-task segmentation framework for thyroid tumor segmentation (Yang et al., 2023).

Inspired by these strategies, it is very promising to obtain a novel method, by incorporating more effective attention mechanisms into U-Net, and using a more appropriate loss function that can further improve the accuracy of FAZ segmentation.

## 2 Related works

The methods for FAZ segmentation of OCTA images are mainly divided into classical methods, traditional machine learning methods and deep learning-based methods.

Among classical methods, the threshold segmentation (Liu et al., 2022) is a simple and commonly used method to segment FAZ based on pixel threshold. Each pixel is compared with a pre-defined threshold, and once the pixel value is greater than the threshold, it is marked as belonging to FAZ. Its segmentation result can be further optimized by subsequent morphological operations. The segmentation method based on region-growing (Ghassemi and Mirzadeh, 2007) utilizes the similarity between seed points and adjacent pixels, where a seed point is first selected, and then the FAZ is gradually expanded by comparing the similarity of adjacent pixels to the seed point. This method requires appropriate similarity measurement and seed point selection. The method based on morphological operation (Yang et al., 2016), such as corrosion, dilation, open and close operations, processes images to extract structures of interest, which achieves a good segmentation effect for objects with obvious morphological features. The frequency domain analysis method (Liu and Li, 2019) is to segment FAZ based on Fourier transform or wavelet transform equal frequency domain analysis technology, which can distinguish between vascular and non-vascular areas by extracting frequency information. The two-stage image processing method proposed by Díaz et al. (2019a) is based on FAZ positioning and contour extraction, which can handle detailed information well. Although these classical methods have made some progress, there are still limitations, such as inaccurate boundary due to poor image quality, confusion between FAZ and non-perfusion region, segmentation error when there is wrong layer projection, and cannot adapt well to complex image scenes and shapes.

In terms of traditional machine learning methods, the method proposed by Simó and Ves (2001) uses a statistical Bayesian segmentation for FAZ detection in digital retinal angiograms, which provides a global segmentation, i.e., veins, arteries and fovea are obtained simultaneously. The method proposed by Alam et al. (2019) employs an AI system containing an SVM classifier model and utilizes a hierarchical backward elimination technique to identify optimal-feature-combination for the best diagnostic accuracy and most efficient classification performance. Another method proposed by Bourennane (2010) first uses singular value decomposition (SVD) to improve signal to noise ratio, then applies MRF for FAZ segmentation, which achieves an encouraging result as a first approach for location and evolution of FAZ in retinal images. These machine learning-based methods on FAZ segmentation usually rely on hand-crafted features and prior knowledge, which are difficult to adapt to the complexity and diversity of FAZ, especially in the segmentation of low-quality images or diseased areas, and are prone to missegmentation or missing segmentation.

Among deep learning-based methods, U-Net (Ronneberger et al., 2015; Sherwani and Gopalakrishnan, 2024) is a landmark network structure for medical image segmentation, which is formed by concatenating feature maps of its encoder branch with feature maps of its decoder branch via skip connections. Subsequently, a variety of improved networks based on this structure are proposed. MED-Net proposed by Guo et al. (2018) is the first deep neural network used for avascular zone detection in OCTA images, which consists of encoders and decoders with multi-scale blocks to capture features at different scales. An automatic superficial FAZ segmentation and quantification method proposed by Guo et al. (2019) to classify each pixel into superficial FAZ or non-superficial FAZ class. Subsequent applied largest connected-region extraction and hole-filling to fine-tune the automatic segmentation results. Another customized encoder-decoder network incorporates a boundary alignment strategy with boundary supervision proposed by Guo et al. (2021) to automatically segment the superficial FAZ. BSDA-Net proposed by Lin et al. (2021) uses boundary regression and distance graph reconstruction of two auxiliary branches to improve the performance of the main branch. A lightweight U-Net proposed by Li et al. (2020) is used to perform fast and robust FAZ segmentation. A segmentation network leveraging optical density and disease features ODDF-Net is proposed by Yang et al. (2024) for the simultaneous 2D segmentation of RC, RA, RV, and FAZ in 3D OCTA, which can learn the relationship between retinal diseases and the disrupted vascular structures, facilitating multi-object structure extraction. A multistage dual-branch image projection network (DIPN) is proposed by Liu et al. (2025) to learn feature information in B-scan images to assist geographic atrophy segmentation and FAZ segmentation. At present, these deep learning-based methods on FAZ segmentation still faces the problems of insufficient segmentation accuracy and limited generalization ability, and still needs to be further improved.

In order to further improve the accuracy of FAZ segmentation while maintaining good generalization ability, we propose a novel improved method named FLA-UNet by incorporating feature attention and location attention into U-Net and using a joint loss function. The main contributions of this paper are as follows:

1. An innovative feature-location attention block (FLAB) is designed by using a feature-aware block and a location-aware block in parallel for each feature map, where the feature-aware block can be used to adjust the weight of each feature map and enhance the expression ability of network, while the location-aware block can obtain the global statistics of each feature map and better retain texture features and background information of FAZ.
2. A novel improved method based on U-Net for FAZ segmentation is proposed by embedding a FLAB into each decoder of U-Net to integrate more marginal information of FAZ and strengthen the connection between target region and boundary information, and using a joint loss function consisting of the cross-entropy loss (CE loss) function and the Dice coefficient loss (Dice loss) function to realize the optimization of the whole continuity of image and the boundary recovery.
3. A series of qualitative and quantitative comparative experiments on the three datasets of OCTAGON, FAZID and OCTA-500 are implemented to show the superiority of our method over other existing state-of-the-art methods in terms of visual segmentation effect and the MIoU, ACC and Dice coefficients.

## 3 Proposed method

Typically, for a basic U-Net structure used for object segmentation, the encoded low-level feature maps are concatenated with the corresponding high-level feature maps from the decoder branch, so the beneficial semantic information and redundant information are simultaneously input to its next layer, which may affect the segmentation accuracy of network. This problem can be solved by adding appropriate attention blocks into the main network. Besides, since the CE loss function used in U-Net is only concerned with the prediction result at pixel level, the generated segmentation boundary may be discontinuous or jagged. This problem can be solved by combining it with the Dice loss function, which is used to measure overlap in segmentation tasks and tends to produce smoother segmentation boundaries, to form a compound loss function to optimize the network model parameters for FAZ segmentation.

### 3.1 Improved network structure

**Figure 1:**
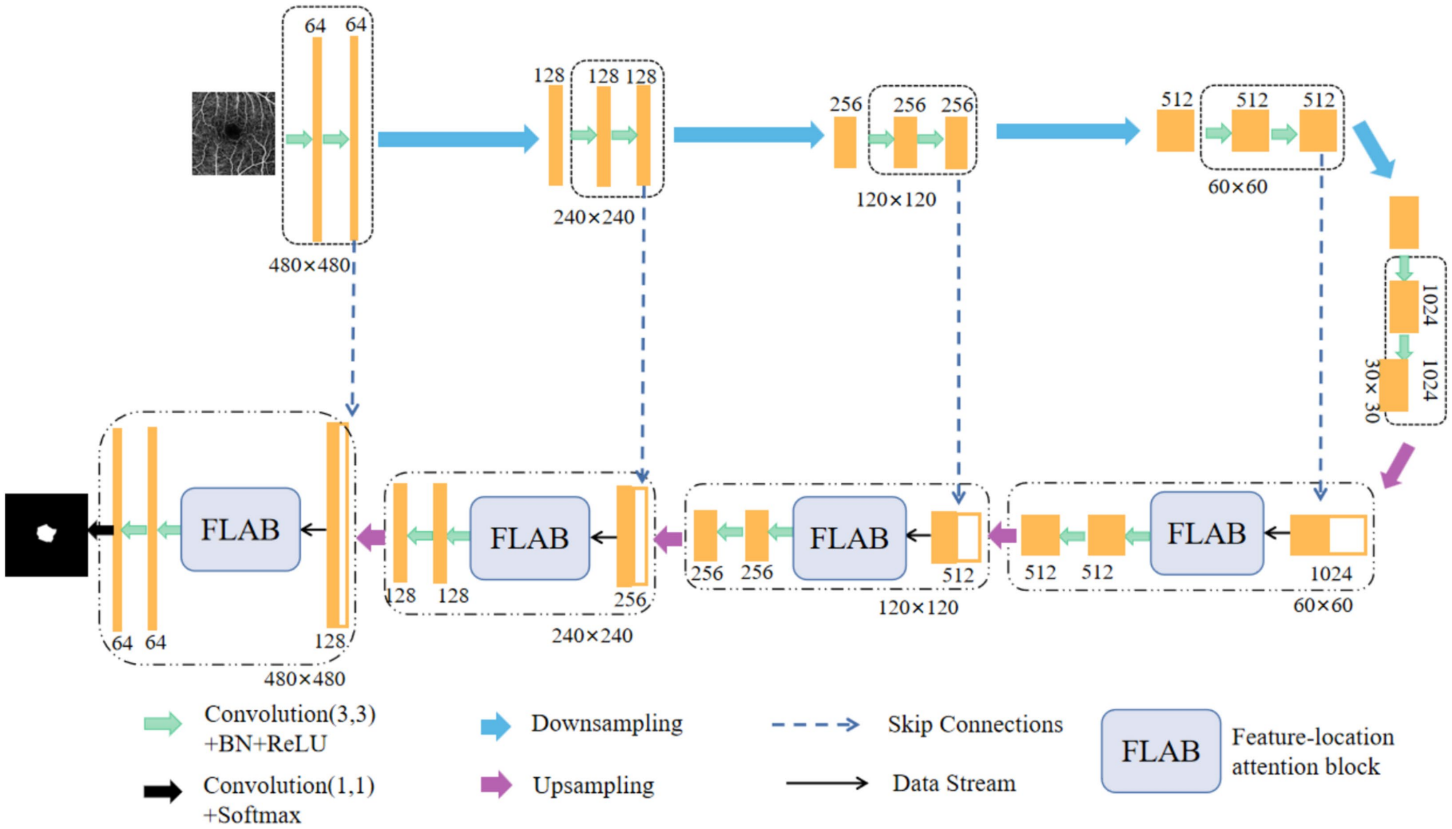
Schematic representation of our improved network structure, which is formed by embedding a FLAB into each decoder of U-Net.

The novel improved network for FAZ segmentation is designed by embedding an innovative FLAB into each decoder of U-Net, as shown in Figure 1. In the encoder branch, five encoders are used to extract features of the input image. Each encoder contains two identical convolution blocks, and each of which consists of a 3 × 3 convolution layer, a batch normalization (BN) layer, and a ReLU activation layer. Between every two encoders, a max pooling operation is used to implement downsampling, and eventually, the spatial dimension is halved by setting the value of stride length to 2 and the number of channels doubles by setting the number of output channels to twice the number of input channels. In the decoder branch, four decoders use the feature maps of encoders to progressively obtain the segmentation result. Each decoder contains a skip connection block, a FLAB, and two identical convolution blocks, each of which is the same as in its corresponding encoder. Between every two decoders (or the last encoder and the first decoder), the bilinear interpolation is used to implement upsampling, where low-resolution feature maps are upsampled to the same resolution as the encoder stage for feature fusion, which can accelerate the training speed of model and make the marginal contour clearer. In each skip connection block, the upsampled feature maps from the previous decoder (or the last encoder) are concatenated with the encoded feature maps from the encoder at current layer, and the concatenated feature maps are input into the corresponding FLAB. In each FLAB, the concatenated feature maps are processed to obtain more detailed features. Finally, the segmentation result is obtained by performing a 1 × 1 convolution operation on the output of the last level decoder, followed by using a Softmax function.

### 3.2 Feature-location attention block

**Figure 2:**
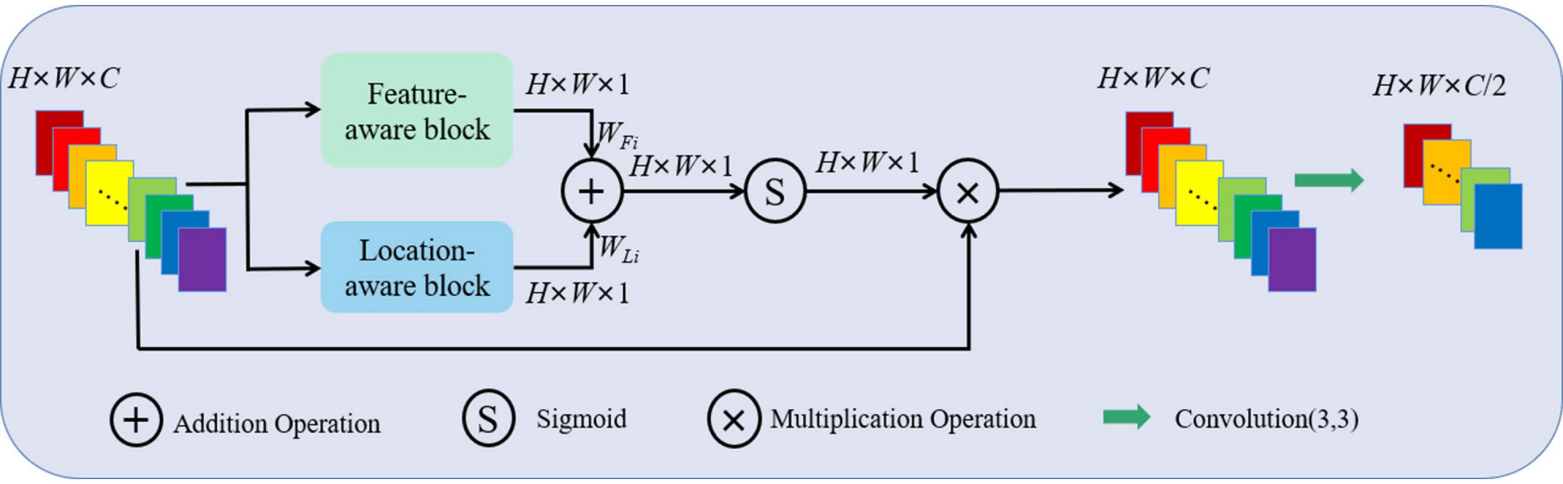
Schematic representation of a FLAB, which mainly consists of a feature-aware block and a location-aware block in parallel.

For the concatenated feature maps *F* ∈ℝ^*H* × *W* × *C*^, a FLAB contains *C* attention modules to process *C* feature maps separately, each attention module consists of a feature-aware block and a location-aware block in parallel, as shown in Figure 2. For channel *i* (*i* ∈1, …, *C*), firstly, the feature weight *W_Fi_* ∈ℝ^*H* × *W* × 1^ and location weight *W_Li_* ∈ℝ^*H* × *W* × 1^ is calculated simultaneously to, respectively, represent the important features in channel and different spatial positions. Then, *W_Fi_* and *W_Li_* are fused together through a simple addition operation to ensure information interaction. Finally, the fused feature map is activated by a Sigmoid function and multiplied with the concatenated feature map in channel *i* to obtain an updated feature map with enhanced feature and location information. The updated feature maps from *C* channels are processed by performing a 3 × 3 convolution operation and halving the number of channels to serve as input of the subsequent convolution block.

For the concatenated feature map *F_i_* ∈ℝ^*H* × *W* × 1^ in channel *i*, firstly, an average pooling and max pooling operation is separately performed in the feature-aware block to produce the feature maps *F*_Avg_∈ℝ^*H* × *W* × 1^ and *F*_Max_∈ℝ^*H* × *W* × 1^. Then, *F*_Avg_ and *F*_Max_ are concatenated to preserve the texture and marginal features of the image. Finally, the feature map sequentially passes through a 1 × 1 convolution layer, a ReLU activation layer and a 1 × 1 convolution layer to generate the feature weight *W_Fi_* ∈ℝ^*H* × *W* × 1^. Its form is shown in Equation 1.

(1) $$W_{\mathrm{Fi}}={\text{conv}}_{1\times 1}(\text{ReLU}({\text{conv}}_{1\times 1}(\text{concat}(F_{\mathrm{Avg}},F_{\mathrm{Max}}))))$$

In the location-aware block, an average pooling and max pooling operation is separately performed on the concatenated feature map *F_i_* ∈ℝ^*H* × *W* × 1^ in channel *i*, and the corresponding feature maps *F*_Avg_∈ℝ^*H* × *W* × 1^ and *F*_Max_∈ℝ^*H* × *W* × 1^ are concatenated similarly. Then, the feature map passes through a 7 × 7 convolution layer to capture the contextual information on location. The output of the convolution layer is the location weight *W_Li_* ∈ℝ^*H* × *W* × 1^. Its form is shown in Equation 2.

(2) $$W_{\mathrm{Li}}={\text{conv}}_{7\times 7}(\text{concat}(F_{\mathrm{Avg}},F_{\mathrm{Max}}))$$

(3) $$W=W_{\mathrm{Fi}}+W_{\mathrm{Li}}$$

Finally, *W_Fi_* and *W_Li_* are fused together through a simple addition operation to ensure information interaction, as shown in Equation 3.

### 3.3 Model optimization and implementation details

In order to optimize the parameters of improved network model for FAZ segmentation, the joint loss function *L*_Jloss_ (Jia et al., 2023; Yang et al., 2023) is adopted, which includes the CE loss function *L*_CE_ and the Dice loss function *L*_Dice_, as shown in Equation 4.

(4) $$L_{\text{Jloss}}=w_{1}L_{\text{Dice}}+w_{2}L_{\mathrm{CE}}$$

Where, *w*_1_ and *w*_2_ are the weight coefficients, *L*_CE_ is used to promote the improved model to learn more accurate classification information and improve its generalization ability, while *L*_Dice_ is used to help it learn more accurate boundary segmentation information, so as to improve the segmentation accuracy. By combining these two loss functions, the robustness of the model and the accuracy of segmentation can be enhanced.

According to the experimental results, *w*_1_ is set to 0.8 and *w*_2_ is set to 0.2. *L*_CE_ and *L*_Dice_ can be expressed by the following Equations 5 and 6.

(5) $$L_{\mathrm{CE}}=-[y\log p+(1-y)\log(1-p)]$$

(6) $$L_{\text{Dice}}=1-2\times \frac{|X\cap Y|}{|X|+|Y|}$$

Where, *y* is the true label, representing the category of the sample, *p* is the prediction probability that the sample belongs to the positive class; *X* represents the positive pixel set in the prediction segmentation image and *Y* represents the positive pixel set in the real segmentation image, *|X|* and *|Y|* respectively indicate the size of the pixel set, while *|X ∩ Y|* represents the intersection size of two-pixel sets.

The proposed FLA-UNet is implemented with Pytorch framework using the NVIDIA A40 on Ubuntu, which has 48 GB memory and 19.5 TFLOPs. The Adam optimizer is used with a learning rate of 0.01, and the model is trained for 200 epochs with a batch size of 8. Each original image is cropped to a size of 480 × 480 for model training. The ratio between the training set and testing set is 7:3. Each dataset is trained three times, and the final model is determined to be the model that has the optimal value of the selected performance metrics on the testing set.

## 4 Experiments and results

### 4.1 Datasets and evaluation metrics

**Figure 3:**
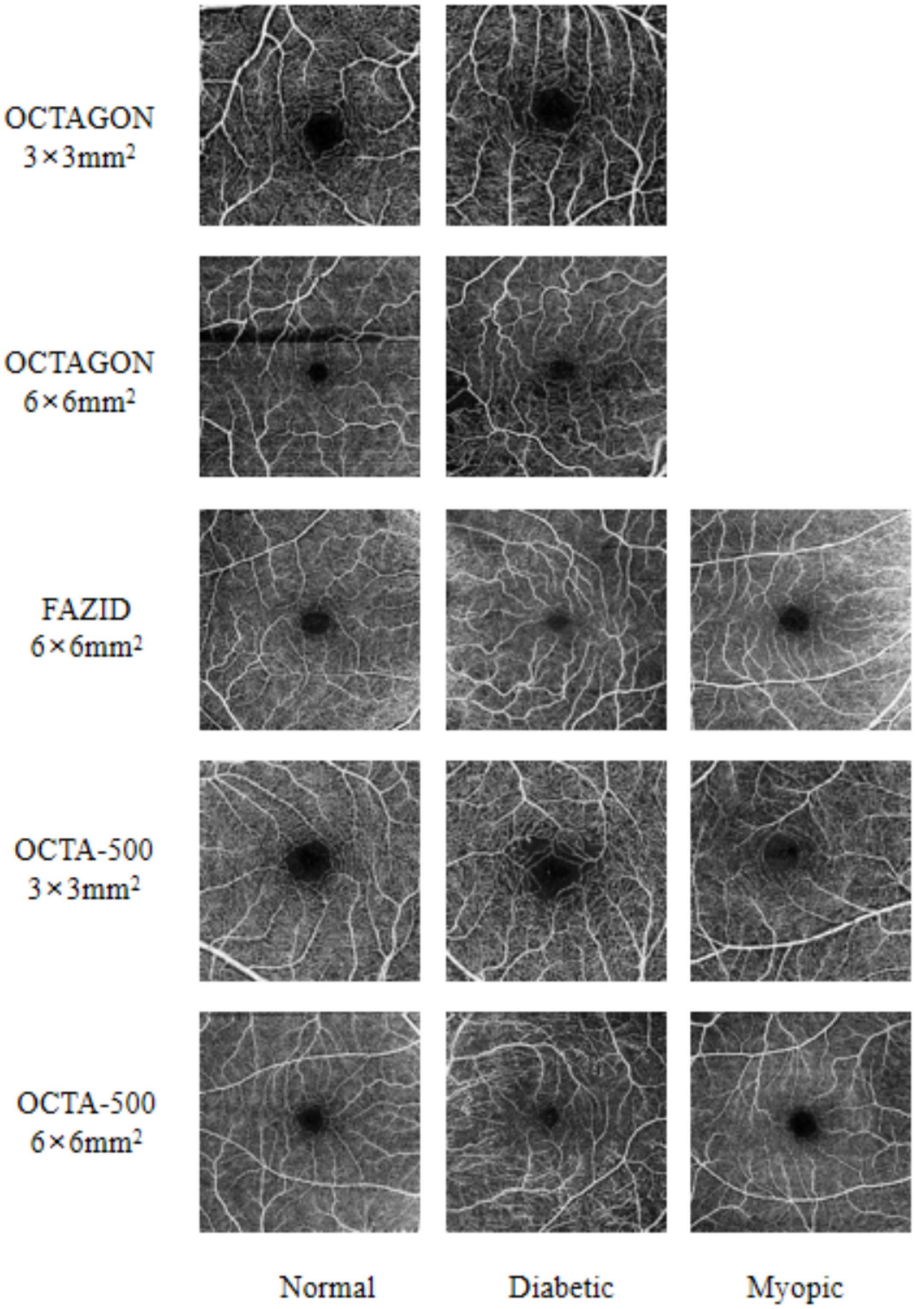
Some sample images from three selected datasets. The FAZ state is listed at the bottom of each column. On the left side of each row of images, the dataset where the picture is located and the field of view are marked.

In order to verify the performance of the proposed FLA-UNet, three public datasets OCTAGON (Díaz et al., 2019b), FAZID (Agarwal et al., 2020) and OCTA-500 (Li et al., 2020) with high image quality are selected, and their details are listed in Table 1. OCTAGON contains 213 OCTA images with a resolution of 320 × 320, 144 of which are *normal* with a field of view (FOV) size of 6 × 6 mm^2^, and 69 of which are *diabetic* with a FOV size of 3 × 3 mm^2^. FAZID consists of 304 OCTA images with a resolution of 420 × 420, 88 of which are *normal*, 109 of which are *myopic* and 107 of which are *diabetic*. All of these OCTA images in FAZID have a FOV size of 6 × 6 mm^2^. For OCTA-500, only three states of images are selected, which are *normal*, *myopic* and *diabetic*. These images are divided into two sub-datasets based on different resolutions and FOV sizes. In the sub-dataset with a resolution of 400 × 400 and a FOV size of 6 × 6 mm^2^, there are 169 OCTA images, 91 of which are *normal*, 43 of which are *myopic* and 35 of which are *diabetic*. While in the sub-dataset with a resolution of 304 × 304 and a FOV size of 3 × 3 mm^2^, there are 195 OCTA images, 160 of which are *normal*, only 6 of which are *myopic* and 29 of which are *diabetic*. The corresponding sample images are shown in Figure 3.

**Table 1:** The details of three selected datasets.

Dataset	FOV [mm^2^]	State	Number		Resolution
OCTAGON	6 × 6	Normal	144	213	320 × 320
	3 × 3	Diabetic	69		
FAZID	6 × 6	Normal	88	304	420 × 420
		Myopic	109		
		Diabetic	107		
OCTA-500	6 × 6	Normal	91	169	400 × 400
		Myopic	43		
		Diabetic	35		
	3 × 3	Normal	160	195	304 × 304
		Myopic	6		
		Diabetic	29		

The quantitative evaluation metrics used for FAZ segmentation are Mean Intersection over Union (MIoU), Accuracy (ACC) and Dice coefficient (Dice), which are defined in Equations 6–9.

(7) $$\text{MIoU}=\frac{1}{k+1}\sum_{i=0}^{k}\frac{\mathrm{TP}}{\mathrm{FN}+\mathrm{FP}+\mathrm{TP}}$$

(8) $$\mathrm{ACC}=\frac{\mathrm{TP}+\mathrm{TN}}{\mathrm{TP}+\mathrm{FP}+\mathrm{TN}+\mathrm{FN}}$$

(9) $$\text{Dice}=\frac{2\mathrm{TP}}{2\mathrm{TP}+\mathrm{FP}+\mathrm{FN}}$$

Where *TP*, *TN*, *FP* and *FN* represent the numbers of true positive, true negative, false positive and false negative pixels respectively, *k* represents the number of segmentation categories, which is set to 1.

### 4.2 Qualitative comparison results

The comparative experiments with the existing state-of-the-art methods (Ronneberger et al., 2015; Gu et al., 2019; Mou et al., 2021; Zhou et al., 2020; Huang et al., 2020; Li et al., 2020; Hu et al., 2022; Li et al., 2022) are carried out to prove the superiority of our proposed method.

**Figure 4:**
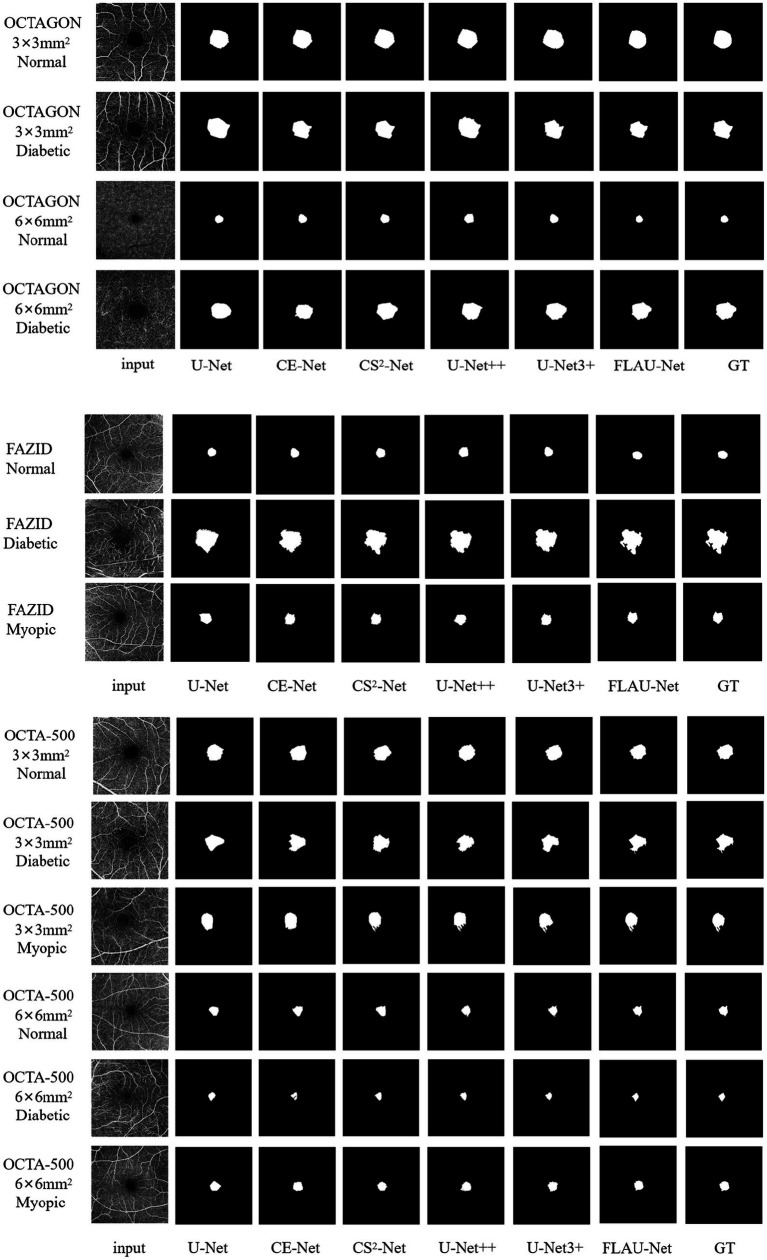
Qualitative comparison of segmentation results using different methods. The left-most and right-most columns, respectively, correspond to the input images and their given GT images. The name at the bottom of each column (except for the left-most and right-most columns) refers to the used segmentation method. On the left side of each row of images, the dataset where the picture is located, the field of view and the FAZ state are marked.

The segmentation results of some examples using the existing representative methods [including the U-Net (Ronneberger et al., 2015), CE-Net (Gu et al., 2019), CS^2^-Net (Mou et al., 2021), U-Net++ (Zhou et al., 2020) and U-Net3 + (Huang et al., 2020)] and our method are shown in Figure 4. We can see that U-Net just roughly segment the outline of FAZ, it is difficult for the basic U-Net to accurately segment the FAZ with irregular contours for myopic and diabetic patients. Despite the good segmentation result on the sample image from OCTAGON, where some sharp regions are also well segmented, CE-Net cannot segment the outline of FAZ well on the sample images from OCTA-500. The similar segmentation effect appears in CS^2^-Net. For U-Net++ and U-Net3+, the outlines of FAZ are affected by blood vessels, resulting in imprecise segmentation. Compared with the above segmentation results, our results have clearer outlines or margins, and are more similar to GT.

### 4.3 Quantitative comparison results

The quantitative comparison results with the existing representative methods on OCTAGON, FAZID and OCTA-500 are shown in Table 2. We can see from Table 2, our method achieves the best segmentation performance on the first two datasets. The MIoU, ACC and Dice of our method on OCTAGON (3 × 3) is, respectively, 1.23, 1.51 and 0.8% higher than results of the suboptimal method. Similarly, the MIoU, ACC and Dice of our method on FAZID is, respectively, 0.55, 2.93 and 1.19% higher than results of the suboptimal method. Although the ACC of our method on OCTAGON (6 × 6) is 1.56% lower than that of U-Net3+, its MIoU and Dice is, respectively, 2.63 and 1.54% higher. On OCTA500 (3 × 3), although both the MIoU and Dice of our method are lower than those of U-Net3+, the differences are small and the ACC of our method is still the highest. On OCTA500 (6 × 6), in spite of the slightly lower MIoU than the result of U-Net++, our method still achieves the highest ACC, and its Dice is 4.17% higher than that of the suboptimal method.

**Table 2:** Quantitative comparisons with the existing representative methods for FAZ segmentation.

Dataset	FOV	Metrics	U-Net (Ronneberger et al., 2015)	CE-Net (Gu et al., 2019)	CS^2^-Net (Mou et al., 2021)	U-Net++ (Zhou et al., 2020)	U-Net3 + (Huang et al., 2020)	FLA-UNet
OCTAGON	3 × 3	MIoU	80.29	74.59	75.31	82.56	83.05	**84.28**
		ACC	95.31	87.57	91.38	95.03	96.14	**97.65**
		Dice	86.16	84.24	84.57	86.52	87.43	**88.23**
	6 × 6	MIoU	81.04	77.49	73.65	80.92	83.83	**86.46**
		ACC	94.91	84.37	92.16	93.05	**96.29**	94.73
		Dice	86.13	85.23	82.64	86.60	87.27	**88.81**
FAZID	6 × 6	MIoU	77.23	78.79	79.14	77.80	79.61	**80.16**
		ACC	87.66	87.39	89.74	88.36	90.15	**93.08**
		Dice	87.22	84.68	83.69	86.55	87.37	**88.56**
OCTA-500	3 × 3	MIoU	79.21	80.22	80.83	81.31	**83.70**	83.11
		ACC	87.51	84.35	83.86	87.76	88.16	**89.31**
		Dice	89.56	87.36	89.12	91.98	**95.22**	93.27
	6 × 6	MIoU	76.88	80.93	79.31	**81.79**	80.16	81.55
		ACC	85.42	83.39	81.71	86.32	87.50	**88.64**
		Dice	87.70	84.68	83.69	88.58	87.44	**92.75**

The best results are marked in bold.

In order to further demonstrate the superiority of our proposed method, we select some recent methods [including Automatic segmentation (Li et al., 2020), Joint-Seg (Hu et al., 2022), and RPS-Net (Li et al., 2022)] for comparison. Due to its universality and importance in medical image segmentation task, the Dice is selected as the indicator for further comparison. The comparison results on OCTAGON and OCTA-500 (6 × 6) are shown in Table 3. As can be seen from Table 3, our method achieves the highest Dice on all three datasets, which confirms its superiority over the selected recent methods.

**Table 3:** The Dice coefficients of quantitative comparisons with the recent methods for FAZ segmentation.

Method	OCTAON		FAZID	OCTA-500	
	3 × 3	6 × 6	6 × 6	3 × 3	6 × 6
Automatic segmentation (Li et al., 2020)	85.00	83.62	85.15	88.64	85.21
Joint-Seg (Hu et al., 2022)	73.25	75.17	74.29	87.01	90.29
RPS-Net (Li et al., 2022)	87.47	86.61	84.98	84.00	91.68
FLA-UNet	**88.23**	**88.81**	**88.56**	**89.31**	**92.75**

### 4.4 Ablation studies

To demonstrate the effectiveness of FLABs and joint loss function used in our proposed method for FAZ segmentation, a series of ablation experiments are conducted. The results of quantitative comparisons for different ablation methods on three datasets are shown in Table 4.

**Table 4:** Quantitative results of different ablation methods for FAZ segmentation.

Dataset	FOV	Metrics	U-Net	U-Net + FLABs	FLA-UNet
OCTAGON	3 × 3	MIoU	80.29	81.24	**84.28**
		ACC	95.31	**98.29**	97.65
		Dice	86.16	87.38	**88.23**
	6 × 6	MIoU	81.04	83.47	**86.46**
		ACC	94.91	**95.75**	94.73
		Dice	86.13	87.62	**88.81**
FAZID	6 × 6	MIoU	77.23	79.65	**80.16**
		ACC	87.66	92.11	**93.08**
		Dice	87.22	88.31	**88.56**
OCTA-500	3 × 3	MIoU	79.21	80.26	**83.11**
		ACC	87.51	87.41	**89.31**
		Dice	89.56	91.46	**93.27**
	6 × 6	MIoU	76.88	80.52	**81.55**
		ACC	85.42	86.37	**88.64**
		Dice	87.70	91.77	**92.75**

As can be seen from Table 4, when FLABs are added in U-Net, the MIoU, ACC and Dice are improved in most cases. This proves that the strategy of introducing FLABs into U-Net is effective. When we further use the joint loss function to adjust the influence of the CE loss function and the Dice loss function, the MIoU, ACC and Dice are further improved in comparison with U-Net + FLABs in most cases. In terms of MIoU and Dice, our proposed FLA-UNet achieves the best performance on three datasets. Although the ACC of our proposed FLA-UNet is not the best on OCTAGON, it achieves the highest values on other two datasets. This proves that the strategy of using the joint loss function also helps to improve the segmentation accuracy.

Based on the above analysis, we can confirm that FLABs and joint loss function are effective, and without them, the model’s segmentation accuracy will deteriorate.

## 5 Conclusion

**Figure 5:**
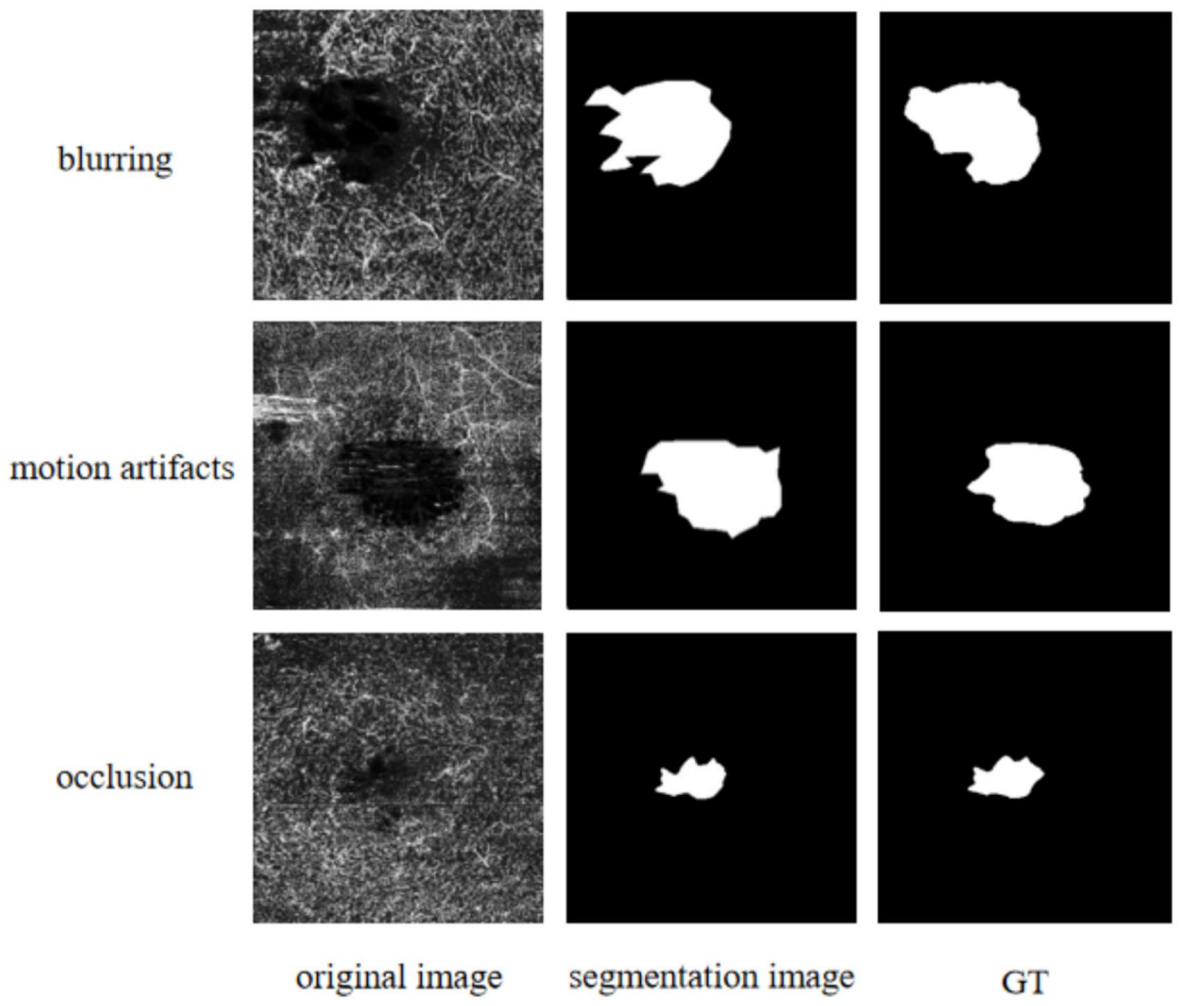
Qualitative results using our proposed FLA-UNet. The left-most column from top to bottom corresponds to the input images, respectively, under the conditions of blur, motion artifacts and occlusion. The middle column refers to the segmentation results using our proposed FLA-UNet. The right-most column refers to their given GT images from OCTAGON.

In this paper, a novel improved method named FLA-UNet is proposed for FAZ segmentation in OCTA images. On the basis of U-Net, by embedding an innovative FLAB into each decoder, the FAZ boundaries are accurately predicted; and by using the joint loss function, the optimization of the whole continuity of an image and its boundary recovery are realized. The effectiveness of FLABs and joint loss function used in FLA-UNet is verified by a series of ablation experiments conducted on OCTAGON, FAZID and OCTA-500. The quantitative comparisons with the existing representative methods on the three datasets show that our proposed FLA-UNet is superior to other methods, in most cases in terms of the MIoU, ACC and Dice coefficient. Accordingly, their qualitative comparison results also confirm this point. In addition, further quantitative comparisons with some recent methods also demonstrate the superiority of our proposed FLA-UNet. It is worth noting that since the OCTA images may be affected by eye movement, improper device parameter setting or ocular lesions of patients, which leads to blur, motion artifacts and occlusion in the images, the input images may have poor quality, as shown in the left-most column in Figure 5. Although our proposed FLA-UNet can segment FAZ to a certain extent, there is still a significant difference between the segmentation results and their GT values, which will lead to some problems in clinical application. In further work, we will try to perform data preprocessing on the input image to enhance the edge contrast between FAZ and its background, to improve the accuracy and reliability of segmentation. Furthermore, the optimization and adjustment of the loss function will also be attempted, such as introducing different train losses commonly used for non-medical applications (Sherwani et al., 2020), to enhance the robustness and generalization of the model. It is believed that, the optimization and application of our proposed FLA-UNet for FAZ segmentation will improve the accuracy of auxiliary diagnosis of fundus diseases.

## Data Availability

Publicly available datasets were analyzed in this study. This data can be found at: https://ieee-dataport.org/open-access/octa-500. The source code is available at: https://github.com/LiCao-WHPU/FLA-UNet.
